# 1264. *In Vitro* Activity of Ceftazidime-Avibactam and Comparator Agents Against Enterobacterales and *Pseudomonas aeruginosa* Collected < 48 Hours and ≥48 Hours Post-Admission from Pediatric Patients, ATLAS Surveillance Program 2016-2019

**DOI:** 10.1093/ofid/ofab466.1456

**Published:** 2021-12-04

**Authors:** Krystyna Kazmierczak, Sibylle Lob, Gregory Stone, Daniel F Sahm

**Affiliations:** 1 IHMA, Inc., Schaumburg, Illinois; 2 Pfizer, Inc., Groton, CT

## Abstract

**Background:**

Ceftazidime-avibactam (CAZ-AVI) is a β-lactam/non-β-lactam β-lactamase inhibitor combination with *in vitro* activity against Enterobacterales (Ent) and *Pseudomonas aeruginosa* (*Psa*) carrying Class A, C and some Class D β-lactamases. We examined the *in vitro* activity of CAZ-AVI and comparators against presumed community-acquired (CA; cultured < 48 h after hospital admission) and hospital-acquired (HA; cultured ≥48 h post-admission) isolates collected from pediatric patients as part of the ATLAS surveillance program.

**Methods:**

6654 non-duplicate isolates were collected in 52 countries in Europe (n=3423), Latin America (n=1323), Middle East/Africa (n=1177), and Asia/Pacific (excluding China; n=731) from patients (newborn to 17 y) with lower respiratory tract (LRTI; n=1687), urinary tract (UTI; n=1631), bloodstream (BSI; n=1149), skin and soft tissue (SSTI; n=1122), and intra-abdominal (IAI; n=981) infections. Susceptibility testing was performed by CLSI broth microdilution and values were interpreted using CLSI 2021 breakpoints. CAZ-AVI was tested at a fixed concentration of 4 µg/mL AVI. Isolates with CAZ or aztreonam MICs ≥2 µg/mL (*Escherichia coli*, *Klebsiella* spp., *Proteus mirabilis*) or meropenem MICs ≥2 µg/mL (all Ent species) or ≥4 µg/mL (*Psa*) were screened for β-lactamase genes.

**Results:**

The *in vitro* activity of CAZ-AVI exceeded that of meropenem and other tested β-lactams against Ent (97.8% susceptible (S)) and *Psa* (92.1% S) collected globally from pediatric patients (Table). Percentages of susceptibility to CAZ-AVI ranged from 95.4-99.2% among CA Ent from different infection types and were reduced 0.6-1.3% among HA isolates from LRTI, UTI, SSTI, and IAI. Susceptibility to CAZ-AVI was also similar (92.6-95.8% S) among CA *Psa* from different infection types and was reduced 1.2-7.0% among HA isolates. Larger differences in susceptibility were typically seen for the tested comparator β-lactams. For Ent, the lowest percentages of susceptibility to the tested β-lactams were observed among isolates from BSI, while the pattern was less clear for *Psa*.

Results Table

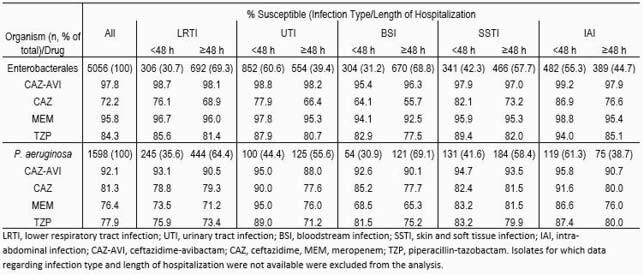

**Conclusion:**

CAZ-AVI could provide a valuable therapeutic option for treatment of CA and HA infections caused by Ent and *Psa* in pediatric patients.

**Disclosures:**

**Krystyna Kazmierczak, PhD**, **IHMA** (Employee)**Pfizer, Inc.** (Independent Contractor) **Sibylle Lob, PhD**, **IHMA** (Employee)**Pfizer, Inc.** (Independent Contractor) **Gregory Stone, PhD**, **AztraZeneca** (Shareholder, Former Employee)**Pfizer, Inc.** (Employee) **Daniel F. Sahm, PhD**, **IHMA** (Employee)**Pfizer, Inc.** (Independent Contractor)

